# Differences in disease activity in cryopyrin-associated periodic syndrome in mutation-positive and mutation-negative patients

**DOI:** 10.1186/1546-0096-13-S1-P69

**Published:** 2015-09-28

**Authors:** M Kostik, L Snegireva, I Babikova, E Kalashnikova, A Rakhimyanova, G Glazyrina, T Knyazeva, L Richkova, V Chasnyk

**Affiliations:** 1 Saint-Petersburg State Pediatric Medical University, Hospital Pediatry, Saint-Petersburg, Russian Federation; 2 Northern State Medical University, Pediatric Department, Arkhangelsk, Russian Federation; 3 Perm Regional children's clinical hospital, Pediatric Rheumatology, Perm, Russian Federation; 4 Regional Children's Hospital №1, Pediatric Rheumatology, Ekaterinburg, Russian Federation; 5 Regional Children's Hospital, Pediatric Rheumatology, Chelyabinsk, Russian Federation; 6 Scientific Center of Family Health and Human Reproduction Problems. Siberian Branch of the Russian Academy of Medical Sciencesuts, Pediatric Department, Irkutsk, Russian Federation

## Introduction

Cryopyrin-associated periodic syndrome (CAPS) is an inherited disease which is caused by gain-of function mutations in CIAS1 gene function resulting in increased secretion of active IL-1β. As known more than 40% of patients with CAPS are genetic negative in CIAS1 gene. One of theories explains this is a fact is a somatic mozaicism.

## Objective

The aim of our study was compare activity of CAPS patients depends on presence or absence of mutations in CIAS1 gene.

## Materials

9 patients with CAPS (6 CINCA and 3 Muckle-Wells syndromes- MWS) were included in our study. In all patients genetic tests in CIAS1 gene was performed. 4 patients have positive mutations (3 CINCA and 1 MWS) and 5 have not any mutations in CIAS1 gene (3 CINCA and 2 MWS). Disease activity was measured with applying simplified auto-inflammatory disease activity index (sAIDAI, M.Piram et al, 2013), MDVAS, levels of erythrocyte sedimentation rate (ESR), c-reactive protein (CRP), hemoglobin (Hb), white blood cells (WBC), platelets (PLT), and fibrinogen. All patients were treated with canakinumab. MDVAS and sAIDAI were evaluated at twice: before canakinumab and during the treatment.

## Results

We have not detected differences in laboratorial markers, such as ESR, CRP, Hb, WBC, PLT and fibrinogen depends on the presence of the CIAS1 mutation, but in mutation positive patients WBC was 2 times higher (n.s.), PLT and fibrinogen was +25% higher than in genetic negative patients. No differences in MDVAS before and on canakinumab, but we have detected differences in sAIDAI before canakinumab: 88.5 (78.5 - 106.5) vs 51.0 (48.0 - 55.0), p=0.014. On canakinumab no differences in sAIDAI depends on the mutation presence. Also mutation-positive patients required higher dosis of canakinumab and shorter intervals between injections.

## Conclusion

Patient with CAPS with mutation in CIAS1 gene have higher inflammatory activity and required more intensive treatment with canakinumab.

